# The SKN‐1/Nrf2 transcription factor can protect against oxidative stress and increase lifespan in *C. elegans* by distinct mechanisms

**DOI:** 10.1111/acel.12627

**Published:** 2017-06-14

**Authors:** Jennifer M.A. Tullet, James W. Green, Catherine Au, Alexandre Benedetto, Maximillian A. Thompson, Emily Clark, Ann F. Gilliat, Adelaide Young, Kathrin Schmeisser, David Gems

**Affiliations:** ^1^ School of Biosciences University of Kent Canterbury Kent CT2 7NZ UK; ^2^ Institute of Healthy Ageing Department of Genetics, Evolution and Environment University College London Gower Street London WC1E 6BT UK; ^3^ Faculty of Health and Medicine Lancaster University Lancaster LA1 4YG UK

**Keywords:** aging, *C. elegans*, oxidative stress, transcription regulation

## Abstract

In *C. elegans,* the *skn‐1* gene encodes a transcription factor that resembles mammalian Nrf2 and activates a detoxification response. *skn‐1* promotes resistance to oxidative stress (Oxr) and also increases lifespan, and it has been suggested that the former causes the latter, consistent with the theory that oxidative damage causes aging. Here, we report that effects of SKN‐1 on Oxr and longevity can be dissociated. We also establish that *skn‐1* expression can be activated by the DAF‐16/FoxO transcription factor, another central regulator of growth, metabolism, and aging. Notably, *skn‐1* is required for Oxr but not increased lifespan resulting from over‐expression of DAF‐16; concomitantly, DAF‐16 over‐expression rescues the short lifespan of *skn‐1* mutants but not their hypersensitivity to oxidative stress. These results suggest that SKN‐1 promotes longevity by a mechanism other than protection against oxidative damage.

## Introduction, Results, Discussion

SKN‐1 is the *C. elegans* functional ortholog of the mammalian Nrf transcription factors. It protects against stress such that deletion or over‐expression of *skn‐1* results in animals that are hypersensitive or resistant, respectively, to stress (Blackwell *et al*., 2015). *skn‐1* also protects against aging: loss of *skn‐1* shortens lifespan and *skn‐1* over‐expression or gain‐of‐function usually increases lifespan (Blackwell *et al*., 2015; Tang & Choe, 2015). As stress resistance and increased lifespan (Age) are often correlated, one possibility is that protection against stress causes longer life (Ristow & Schmeisser, 2011).

Correlated stress resistance and longevity is also observed in worms with reduced insulin/IGF‐1 signaling (rIIS), dependent upon the transcription factor DAF‐16/FoxO (Kenyon, 2010). Results from combined mRNA and chromatin profiling suggest that DAF‐16 acts as a central regulator within a gene network (Schuster *et al*., 2010; Tullet, 2014). Notably, although these predicted direct DAF‐16 targets include few effectors of stress resistance, one of them is *skn‐1* (Schuster *et al*., 2010). Like *daf‐16*,* skn‐1* contributes to rIIS Age and stress resistance (Blackwell *et al*., 2015).

This raises the possibility that activation of *skn‐1* expression by DAF‐16 promotes stress resistance and, consequently, increased lifespan.

**Figure 1:**
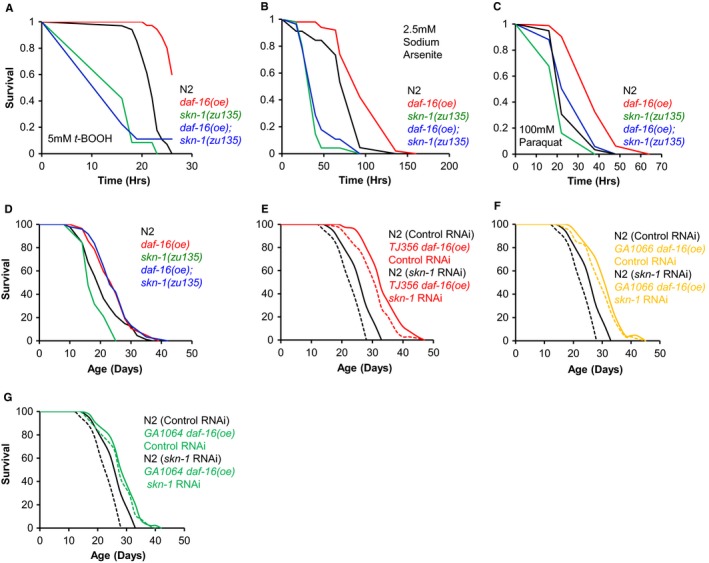
SKN‐1 is required for *daf‐16(oe)* Oxr but not Age. (A–C) *skn‐1* is required for *daf‐16(oe)* Oxr. One representative trial shown in each case. (A) 5 mm *t*‐BOOH. *daf‐16(oe)* increases survival by +14.1%, *P *<* *0.0001 (log rank test; combined data from 3 trials). (B) 2.5 mm sodium arsenite. *daf‐16(oe)* increases survival by +34.1%, *P *<* *0.0001 (log rank test; representative data from 2 trials). (C) 100 mm Paraquat. *daf‐16(oe)* increases survival by +34.2%, *P *<* *0.0001 (log rank test; combined data from 3 trials). (D) *daf‐16(oe)* (*zIs356*) Age does not require SKN‐1 (trial 1 in Table S1). (E,F) *daf‐16(oe)* Age is not suppressed by *skn‐1* RNAi (both trial 1 in Table S1). (G) Age resulting from intestine‐specific *daf‐16(oe)* is not suppressed by *skn‐1* RNAi (trial 1 in Table S1). Assays performed at 20 °C with 40 μm (D) or 80 μm FUDR (E–G).

If *skn‐1* expression is activated by DAF‐16, then SKN‐1 could mediate the phenotypic effects of DAF‐16 activation. *daf‐16* over‐expression (oe) using *zIs356*, a multicopy transgene array, increases resistance to stress (Henderson & Johnson, 2001) and extends lifespan (Qi *et al*., 2012). We therefore assessed whether SKN‐1 is required for *daf‐16(oe)* stress resistance and Age. First, we used *zIs356* to compare the resistance of *daf‐16(oe)* and *daf‐16(oe); skn‐1(zu135)* worms to oxidative stress. *daf‐16(oe)* animals proved to be resistant to *tert*‐butyl hydroperoxide (*t*‐BOOH) and sodium arsenite, and this resistance was dependent on *skn‐1* (Fig. 1A and 1B). Similar results were also obtained with respect to paraquat resistance, although here *skn‐1* only partially suppressed the resistance of *daf‐16(oe)* (Fig. 1C). However, SKN‐1 was dispensable for *daf‐16(oe)* resistance to heat (Fig. S1). Thus, *daf‐16(oe)* wholly or partially requires SKN‐1 to promote Oxr but not thermotolerance.

Next we tested whether SKN‐1 was required for *daf‐16(oe)* Age. *skn‐1(zu135)* alone reduced lifespan but, strikingly, not in *daf‐16(oe)* animals (3 trials; Fig. 1D, Table S1). Thus, the effects of *skn‐1* on *daf‐16(oe)* Age and Oxr are separable. It is striking here that the life‐shortening effects of *skn‐1* are suppressed by *daf‐16(oe)*, even though the worms remain sensitive to oxidative stress. This indicates that the life‐shortening effect of loss of function of *skn‐1* is not due to the concomitant increase in sensitivity to oxidative stress.

We then sought to verify this unexpected conclusion using *skn‐1(RNAi)*, and *daf‐16(oe)* achieved by three means: *zIs356*,* muEx176* (*Pdaf‐16::daf‐16a::gfp*) or *muEx227* (*Pges‐1::daf‐16a::gfp,* intestine‐limited over‐expression) (Libina *et al*., 2003; Alic *et al*., 2014). In most trials, *skn‐1(RNAi)* either did not reduce *daf‐16(oe)* Age (4/7 trials), or it reduced lifespan to a similar extent in N2 and *daf‐16(oe)* populations (2/7 trials; *P *=* *0.46, 0.88; Cox proportional hazard analysis [CPHA]) (Fig. 1E–G, Table S1). In only one trial did *skn‐1(RNAi)* reduce lifespan marginally more in the *daf‐16(oe)* populations (*P *=* *0.04, CPHA). By contrast, *skn‐1(RNAi)* significantly reduced N2 lifespan in all trials (Table S1). Thus, in all RNAi trials, *daf‐16(oe)* either fully or partially suppressed the short lifespan resulting from *skn‐1* RNAi, consistent with results with *skn‐1(zu135)*.

Effects of *daf‐16(oe)* on aging can be masked by premature death associated with *daf‐16(oe)*‐induced germline hyperplasia, but treatment with an inhibitor of DNA replication 5‐fluoro‐2‐deoxyuridine (FUDR) prevents this, unmasking the effect of *daf‐16(oe)* on lifespan (Qi *et al*., 2012). One possibility is that *skn‐1* does not suppress *daf‐16(oe)* longevity because it also rescues the *daf‐16(oe)* germline abnormality. However, *skn‐1* did not alter the frequency of germ cells outside the basal gonad membrane (Fig. S2) arguing against this. Another possibility is that *skn‐1* does not reduce *daf‐16(oe)* lifespan because FUDR suppresses effects of *skn‐1* on lifespan. However, the short lifespan on FUDR of three different *skn‐1* mutants argues against this (Fig. S3A, Table S1).

It is notable that in *daf‐2* mutants, where DAF‐16 is activated, longevity is *skn‐1* dependent, but in *daf‐2*(+)*; daf‐16(oe)* worms it is not. This could imply that *daf‐16(oe)* Age is only SKN‐1 dependent given rIIS. To test this, we compared the life spans of *daf‐16(oe)* and *daf‐16(oe); skn‐1* worms subjected to *daf‐2* RNAi. As expected, *daf‐2* RNAi greatly extended the lifespan of WT worms and this was partially suppressed by *skn‐1* (*P *<* *0.0001) (Tullet *et al*., 2008). *daf‐2* RNAi also increased the lifespan of *daf‐16(oe)* worms but notably this was not suppressed by *skn‐1* (Fig. S3B, Table S1). Unexpectedly, *daf‐16(oe)* reduced the lifespan of worms subjected to *daf‐2* RNAi (*P *<* *0.0001 in each of 2 trials) (Fig. S3B, Table S1), perhaps reflecting excessive DAF‐16 activity. In summary, it is not the case that longevity induced by increased DAF‐16 activity is only SKN‐1 dependent given rIIS. Moreover, *daf‐16(oe)* suppresses the SKN‐1 dependence of rIIS longevity.

A long‐standing theory in the aging field is that aging is caused by accumulated oxidative damage, but some *C. elegans* studies have argued against this (reviewed in Gems & Partridge, 2013). However, SKN‐1 not only promotes longevity but also resistance to pro‐oxidants. If protection against molecular damage promotes *daf‐16(oe)* Age, then our lifespan results could be explained by *daf‐16(oe)* compensating for loss of *skn‐1* by inducing other antioxidant defences. If correct, this predicts that elevation of protein oxidation levels in *skn‐1* mutants should be suppressed by *daf‐16(oe)*. We tested this by measuring protein carbonyl levels in worm protein extracts. Although results were variable, there was a trend toward *skn‐1* worms having increased levels of protein oxidation compared to wild‐type (WT), as seen previously (Rea *et al*., 2007); this was also seen in *daf‐16(oe)* animals (Fig. S4), which is consistent with a previous study (Cabreiro *et al*., 2011). The latter trend was not worsened by *skn‐1(zu135)*, but lessened (Fig. S4). Similar results were also observed in trials using *skn‐1* RNAi (Fig. S4). Thus, *daf‐16(oe)* does not reduce overall levels of protein oxidation in *skn‐1* mutants.

**Figure 2:**
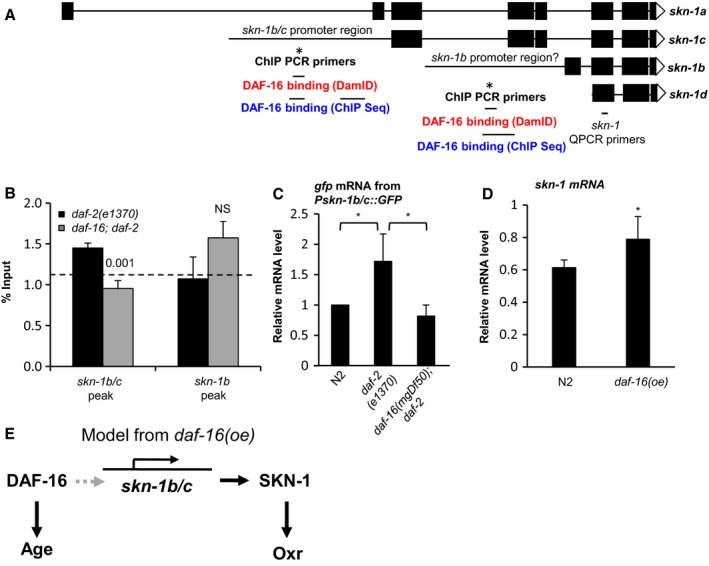
*Pskn‐1b/c* has the capacity for transcriptional activation by DAF‐16/FoxO. (A) Schematic representation of the *skn‐1* locus. This shows the location of putative DAF‐16 binding sites identified by DamID (Schuster *et al*., 2010) and ChIP Seq (Niu *et al*., 2011), and of ChIP PCR primers. (B) DAF‐16 binds to *Pskn‐1b/c* but not *Pskn‐1b*. A DAF‐16‐specific antibody (Santa Cruz) was used for ChIP. The horizontal dotted line indicates % input from a region 5′ of *Pskn‐1b/c* not predicted to bind DAF‐16 (Schuster *et al*., 2010). One representative experiment (of three) is shown which contained 3 IP replicates from the same chromatin preparation (mean ± SD). (C) *daf‐16*‐dependent increase in *gfp* mRNA levels in *Pskn‐1b/c::gfp daf‐2* animals. *P <* *0.05*,* mean ± SD, 3 independent trials. Prior to transgene expression analysis animals were maintained at 15 °C until the L4 stage and then shifted to 25 °C for 24 h. (D) *skn‐1* mRNA level is increased by *daf‐16(oe)* (*zIs356*). *P <* *0.05, mean ± SD, 3 independent trials. (E) Scheme showing the DAF‐16/SKN‐1 portion of the DAF‐16 gene‐regulatory network, based on the *daf‐16(oe)* context where SKN‐1 promotes Oxr but not Age. Dashed arrow denotes context dependent capacity for transcriptional activation from *Pskn‐1b/c* by DAF‐16. Refer to supplement for methods to these and subsequent experiments.

We had previously identified *skn‐1* as potentially bound and transcriptionally activated by DAF‐16 (Schuster *et al*., 2010). To test this further, we verified binding of DAF‐16 to the *skn‐1* promoter, comparing *daf‐2* and *daf‐16; daf‐2* adults using chromatin immunoprecipitation (ChIP) and PCR. Our previous chromatin profiles suggested two DAF‐16 binding sites at the *skn‐1* locus (Fig. 2A). Re‐examining this confirmed DAF‐16 binding to the *skn‐1b/c* promoter (Fig. 2B).

mRNA profile data (microarrays) showed a 2.4‐fold increase in *skn‐1* mRNA (*q *=* *0.09) in *glp‐4(bn2); daf‐2(m577)* relative to *daf‐16(mgDf50) glp‐4; daf‐2* in young adult hermaphrodites (McElwee *et al*., 2007). However, comparison of *skn‐1* mRNA levels in *daf‐2(e1370) vs daf‐16; daf‐2* animals using RT–QPCR did not confirm this (Fig. S5A). As an additional test of DAF‐16 regulation of *skn‐1* expression, we created transgenic worm lines containing a *Pskn‐1b/c::gfp* transcriptional reporter and crossed them into *daf‐2(e1370)* and *daf‐16; daf‐2* backgrounds. In a WT genetic background *Pskn‐1b/c::GFP* was expressed in mesendodermal tissues (Fig. S5B), consistent with the established role of SKN‐1 in development (Blackwell *et al*., 2015) and was broadly similar at all developmental stages. *daf‐2(e1370)* increased expression from this reporter (*gfp* mRNA levels), dependent upon *daf‐16* (Fig. 2C) consistent with direct activation of the *skn‐1b/c* promoter by DAF‐16. GFP fluorescence was not changed by rIIS (Fig. S5C), but this could reflect the global reduction of protein synthesis caused by *daf‐2(e1370)* (Depuydt *et al*., 2013).

To understand these results, we reasoned that *skn‐1* may possess a latent capacity to be up‐regulated by DAF‐16 that becomes detectable using the *skn‐1::GFP* transgene array. This could reflect the increased gene copy number in the array and/or greater stability of *gfp* mRNA (Fig. 2C). Consistent with this interpretation, we detected an increase in *skn‐1* mRNA levels in *daf‐16(oe)* worms relative to WT using RT–PCR (Fig. 2D). Together, these results suggest that by virtue of its DAF‐16 binding site (Schuster *et al*., 2010) *skn‐1* has the capacity to be upregulated by DAF‐16 via binding to the *skn‐1b/c* promoter. However, this capacity remains latent in some contexts in which DAF‐16 activity is increased, and it is possible that it is only manifested under artificial conditions such as *daf‐16* over‐expression.

AMP‐activated protein kinase (AMPK) is, like SKN‐1, required for *daf‐2* longevity and, like *skn‐1*, it is directly up‐regulated by DAF‐16 (Tullet, 2014). AMPK also acts upstream of DAF‐16 (Greer *et al*., 2007). We therefore wondered whether *daf‐16(oe)* Age might also be AMPK independent. Mutation of the AMPK α subunit gene *aak‐2* fully suppresses *daf‐2* longevity (Apfeld *et al*., 2004). However, *aak‐2* did not suppress *daf‐16(oe)* Age (Fig. S3C). That neither SKN‐1 nor AMPK are required for *daf‐16(oe)* Age could imply that DAF‐16 effectors that extend lifespan vary according to whether DAF‐16 activation results from rIIS or *daf‐16(oe)* or, in the case of AMPK, may signify that DAF‐16 activation circumvents the need for AMPK upstream.

To conclude, this study reveals that the effects of SKN‐1 on Oxr and Age can be separated, implying that promotion of longevity by SKN‐1 can act by mechanisms other than oxidative stress resistance (Fig. 2E). This is consistent with the general conclusion that oxidative damage does not play a significant role in aging in *C*. *elegans*. SKN‐1 also transcriptionally regulates genes involved with other processes, for example, autophagy and collagen synthesis and, like IIS, plays a role in early development (Blackwell *et al*., 2015). Identification of the actual effector mechanisms by which SKN‐1 protects against aging is an important future challenge, particularly given the evolutionary conserved role of *skn‐1*/Nrfs in the control of aging (Sykiotis & Bohmann, 2008).

## Funding

Some strains were provided by the Caenorhabditis Genetics Center, which is funded by NIH Office of Research Infrastructure Programs (P40 OD010440). This work was supported by a Wellcome Trust Strategic Award (CA, AFG, DG and JMAT) and a Royal Society Research grant (JMAT).

## Conflict of interest

None declared.

## Supporting information


**Fig. S1** Resistance to heat stress measured in liquid.
**Fig. S2** *skn‐1* mutation does not affect the germline hyperplasia and basal membrane disruption of the germline of *daf‐16(oe)* animals.
**Fig. S3** Tests for interactions between factors affecting lifespan.
**Fig. S4** No difference in protein damage detected in response to *skn‐1* mutation or *skn‐1* RNAi in N2 or *daf‐16(oe)* animals.
**Fig. S5** *skn‐1* mRNA and *Pskn‐1b/c::GFP* fluorescence levels.

**Table S1** Statistics for lifespan measurements.
**Data S1** Supplemental material.

## References

[acel12627-bib-0001] Alic N , Tullet JM , Niccoli T , Broughton S , Hoddinott MP , Slack C , Gems D , Partridge L (2014) Cell‐nonautonomous effects of dFOXO/DAF‐16 in aging. Cell Rep. 6, 608–616. 24508462 10.1016/j.celrep.2014.01.015 PMC3969275

[acel12627-bib-0002] Apfeld J , O'Connor G , McDonagh T , DiStefano PS , Curtis R (2004) The AMP‐activated protein kinase AAK‐2 links energy levels and insulin‐like signals to lifespan in *C. elegans* . Genes Dev. 18, 3004–3009. 15574588 10.1101/gad.1255404 PMC535911

[acel12627-bib-0003] Blackwell TK , Steinbaugh MJ , Hourihan JM , Ewald CY , Isik M (2015) SKN‐1/Nrf, stress responses, and aging in Caenorhabditis elegans. Free Radic. Biol. Med. 88, 290–301. 26232625 10.1016/j.freeradbiomed.2015.06.008 PMC4809198

[acel12627-bib-0004] Cabreiro F , Ackerman D , Doonan R , Araiz C , Back P , Papp D , Braeckman BP , Gems D (2011) Increased life span from overexpression of superoxide dismutase in Caenorhabditis elegans is not caused by decreased oxidative damage. Free Radic. Biol. Med. 51, 1575–1582. 21839827 10.1016/j.freeradbiomed.2011.07.020 PMC3202636

[acel12627-bib-0005] Depuydt G , Xie F , Petyuk VA , Shanmugam N , Smolders A , Dhondt I , Brewer HM , Camp DG , Smith RD , Braeckman BP (2013) Reduced insulin/IGF‐1 signaling and dietary restriction inhibit translation but preserve muscle mass in Caenorhabditis elegans. Mol. Cell Proteomics 12, 3624–3639. 24002365 10.1074/mcp.M113.027383 PMC3861712

[acel12627-bib-0006] Gems D , Partridge L (2013) Genetics of longevity in model organisms: debates and paradigm shifts. Annu. Rev. Physiol. 75, 621–644. 23190075 10.1146/annurev-physiol-030212-183712

[acel12627-bib-0007] Greer EL , Oskoui PR , Banko MR , Maniar JM , Gygi MP , Gygi SP , Brunet A (2007) The energy sensor AMP‐activated protein kinase directly regulates the mammalian FOXO3 transcription factor. J. Biol. Chem. 282, 30107–30119. 17711846 10.1074/jbc.M705325200

[acel12627-bib-0008] Henderson ST , Johnson TE (2001) daf‐16 integrates developmental and environmental inputs to mediate aging in the nematode Caenorhabditis elegans. Curr. Biol. 11, 1975–1980. 11747825 10.1016/s0960-9822(01)00594-2

[acel12627-bib-0009] Kenyon CJ (2010) The genetics of ageing. Nature 464, 504–512. 20336132 10.1038/nature08980

[acel12627-bib-0010] Libina N , Berman JR , Kenyon C (2003) Tissue‐specific activities of *C. elegans* DAF‐16 in the regulation of lifespan. Cell 115, 489–502. 14622602 10.1016/s0092-8674(03)00889-4

[acel12627-bib-0011] McElwee JJ , Schuster E , Blanc E , Piper MD , Thomas JH , Patel DS , Selman C , Withers DJ , Thornton JM , Partridge L , Gems D (2007) Evolutionary conservation of regulated longevity assurance mechanisms. Genome Biol. 8, R132. 17612391 10.1186/gb-2007-8-7-r132 PMC2323215

[acel12627-bib-0012] Niu W , Lu ZJ , Zhong M , Sarov M , Murray JI , Brdlik CM , Janette J , Chen C , Alves P , Preston E , Slightham C , Jiang L , Hyman AA , Kim SK , Waterston RH , Gerstein M , Snyder M , Reinke V (2011) Diverse transcription factor binding features revealed by genome‐wide ChIP‐seq in *C. elegans* . Genome Res. 21, 245–254. 21177963 10.1101/gr.114587.110 PMC3032928

[acel12627-bib-0013] Qi W , Huang X , Neumann‐Haefelin E , Schulze E , Baumeister R (2012) Cell‐nonautonomous signaling of FOXO/DAF‐16 to the stem cells of Caenorhabditis elegans. PLoS Genet. 8, e1002836. 22916022 10.1371/journal.pgen.1002836 PMC3420913

[acel12627-bib-0014] Rea SL , Ventura N , Johnson TE (2007) Relationship between mitochondrial electron transport chain dysfunction, development, and life extension in Caenorhabditis elegans. PLoS Biol. 5, e259. 17914900 10.1371/journal.pbio.0050259 PMC1994989

[acel12627-bib-0015] Ristow M , Schmeisser S (2011) Extending life span by increasing oxidative stress. Free Radic. Biol. Med. 51, 327–336. 21619928 10.1016/j.freeradbiomed.2011.05.010

[acel12627-bib-0016] Schuster E , McElwee JJ , Tullet JM , Doonan R , Matthijssens F , Reece‐Hoyes JS , Hope IA , Vanfleteren JR , Thornton JM , Gems D (2010) DamID in *C. elegans* reveals longevity‐associated targets of DAF‐16/FoxO. Mol. Syst. Biol. 6, 399. 20706209 10.1038/msb.2010.54 PMC2950082

[acel12627-bib-0017] Sykiotis GP , Bohmann D (2008) Keap1/Nrf2 signaling regulates oxidative stress tolerance and lifespan in Drosophila. Dev. Cell 14, 76–85. 18194654 10.1016/j.devcel.2007.12.002 PMC2257869

[acel12627-bib-0018] Tang L , Choe KP (2015) Characterization of skn‐1/wdr‐23 phenotypes in Caenorhabditis elegans; pleitrophy, aging, glutathione, and interactions with other longevity pathways. Mech. Ageing Dev. 149, 88–98. 26056713 10.1016/j.mad.2015.06.001

[acel12627-bib-0019] Tullet JMA (2014) DAF‐16 target identification in *C. elegans*: past, present and future. Biogerontology 16, 221–234. 25156270 10.1007/s10522-014-9527-y PMC4361755

[acel12627-bib-0020] Tullet JM , Hertweck M , An JH , Baker J , Hwang JY , Liu S , Oliveira RP , Baumeister R , Blackwell TK (2008) Direct inhibition of the longevity‐promoting factor SKN‐1 by insulin‐like signaling in *C. elegans* . Cell 132, 1025–1038. 18358814 10.1016/j.cell.2008.01.030 PMC2367249

