# Supplementary figures and images for: The SKN‐1/Nrf2 transcription factor can protect against oxidative stress and increase lifespan in C. elegans by distinct mechanisms
Source: Aging Cell. 2017 Jun 14;16(5):1191–4. doi: 10.1111/acel.12627 (PMC5595692; doi:10.1111/acel.12627)

Figure S1

A  
Resistance to heat in liquid

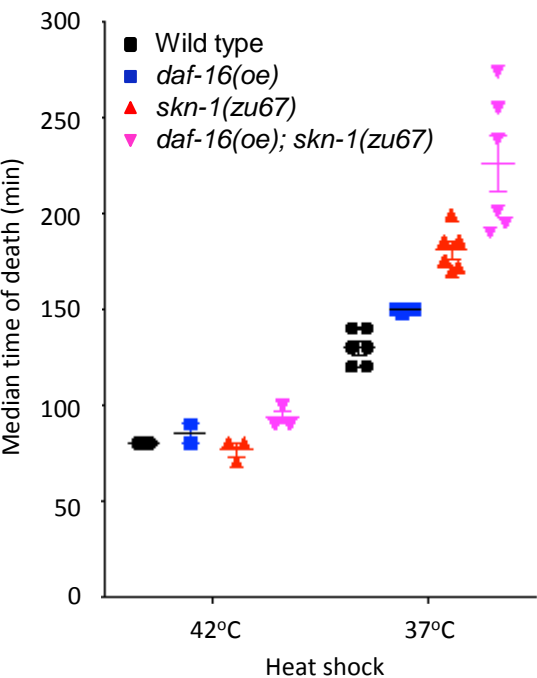

**Figure S2**

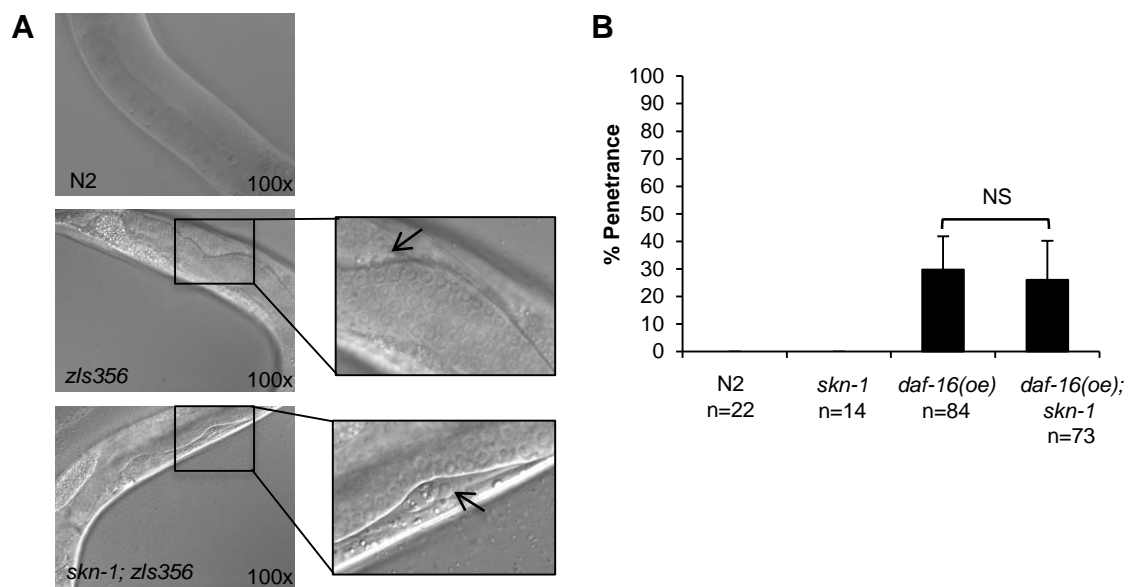

**Figure S3**

**A**

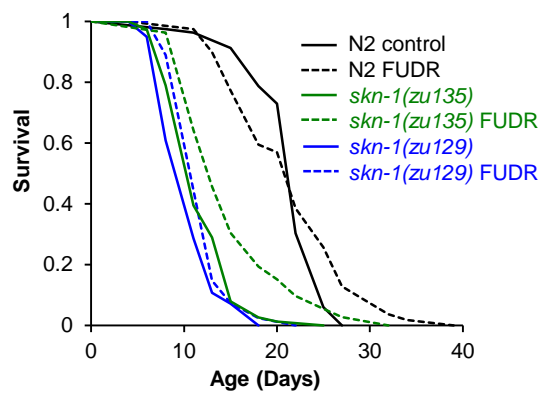

**B**

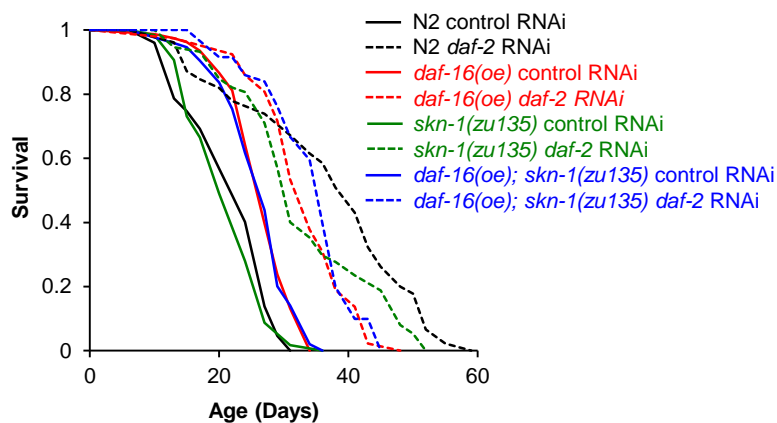

**C**

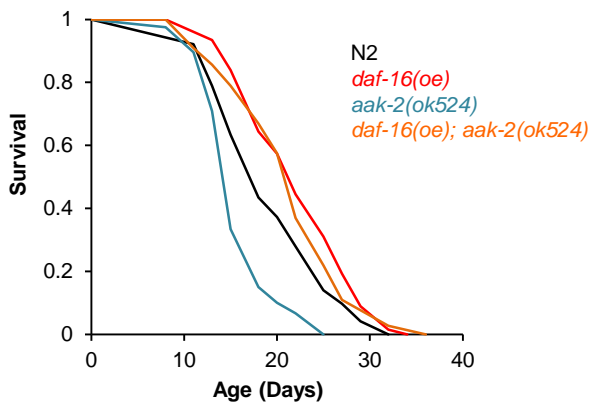

Figure S4

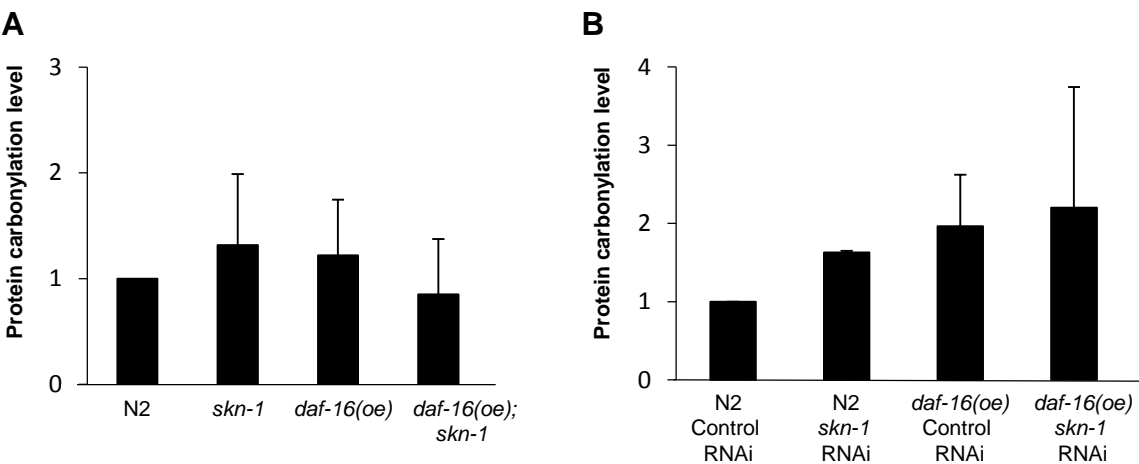

Figure S5

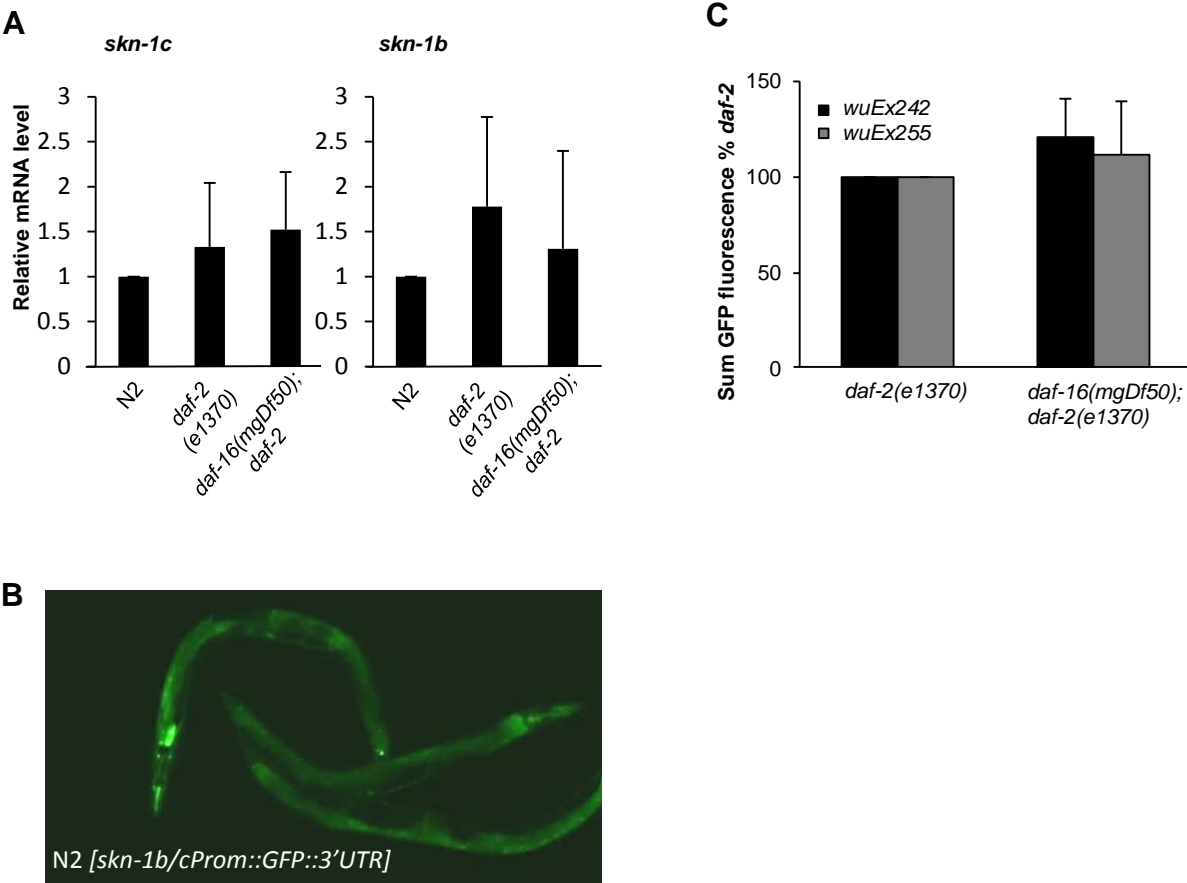

Supplement: Supplementary file 1 — Fig. S1 Resistance to heat stress measured in liquid. Fig. S2 skn‐1 mutation does not affect the germline hyperplasia and basal membrane disruption of the germline of daf‐16(oe) animals. Fig. S3 Tests for interactions between factors affecting lifespan. Fig. S4 No difference in protein damage detected in response to skn‐1 mutation or skn‐1 RNAi in N2 or daf‐16(oe) animals. Fig. S5 skn‐1 mRNA and Pskn‐1b/c::GFP fluorescence levels. [file ACEL-16-1191-s001.pdf]
