# Supplementary material for: The SKN‐1/Nrf2 transcription factor can protect against oxidative stress and increase lifespan in C. elegans by distinct mechanisms
Source: Aging Cell. 2017 Jun 14;16(5):1191–4. doi: 10.1111/acel.12627 (PMC5595692; doi:10.1111/acel.12627)
Supplement: Supplementary file 2 — Table S1 Statistics for lifespan measurements. Data S1 Supplemental material. [file ACEL-16-1191-s002.pdf]

Supplementary material

Strains and cloning

Worms were cultured according as previously described (Brenner 1974), and maintained at 20˚C unless otherwise indicated. The following strains were used: DR1563 *daf-2(e1370),* ESF7 *daf-16(mgDf50); daf-2(e1370),* EU1 *skn-1(zu67),* EU31 *skn-1(zu135),* EU40 *skn-1(zu129),* GA1022 *wuEx242[Pskn-1b/c::GFP],* GA1026 *skn-1(zu135) zIs356 [Pdaf-16::DAF-16a::GFP; rol-6(su1006)]*; *aak-2(ok524),* GA1029 *daf-2(e1370) wuEx242[Pskn-1b/c::GFP],* GA1031 *wuEx255[Pskn-1b/c::GFP],* GA1032 *daf-2(e1370) wuEx255[Pskn-1b/c::GFP],* GA1038 *daf-16(mgDf50); daf-2(e1370) wuEx242[Pskn-1b/c::GFP],* GA1039 *daf-16(mgDf50); daf-2(e1370) wuEx255[Pskn-1b/c::GFP],* GA1056 *aak-2(ok524); zIs356,* GA1064 *muEx227[Pges-1::DAF-16a::GFP; rol-6],* GA1066 *muEx176[Pdaf-16::DAF-16a::GFP; rol-6],* N2 (wild type), and TJ356 *zIs356.* N.B. During the course of this study we tried on 10 occasions to cross the DAF-16 *zIs356* transgene into strains with *skn-1(zu67)* or *skn-1(zu129)*, but no transgenic *skn-1* progeny could be isolated. The genomic insertion site of *zIs356* is unknown, and it is possible that it is close to the *skn-1* locus and therefore difficult to combine with *skn-1* mutant alleles.

promoter::gfp transgenes with *skn-1b/c* were created using Gateway cloning (Invitrogen). The gateway primer sequences that define the region used for *Pskn-1b/c* were: F: ggggacaactttgtatagaaaagttggcatgcactcgccctctcgctacaacatt, R: ggggactgctttttgtacaaacttgccgcggacgcgtctgaaaatatatatca. These were fused to GFP and the endogenous *skn-1* 3’UTR. The transgenic strains were created by microinjection of 50μg of the plasmid with *rol-6(su1006)* as a co-transformational marker, and two independent transgenic lines isolated.

Lifespan and stress assays

Prior to experiments animals were maintained at the permissive temperature and grown for at least two generations with ample *E. coli* food source to assure full viability. Lifespan assays were performed essentially as described (Hsin & Kenyon 1999). Survival plots and statistical comparisons (log rank test) were performed using JMP software, version 7. For lifespan assays using RNAi, worms were grown on bacteria expressing the appropriate RNAi clone from the L4 stage. *E. coli* HT115 bearing the empty pL4440 vector was used as a control. For stress resistance assays young adult worms were challenged either with *t*-BOOH, sodium arsenite or paraquat and survival scored at regular intervals as described (Tullet et al. 2008).

Heat stress assays were carried out in liquid culture using death fluorescence as a readout of mortality (Coburn et al. 2013); a detailed account of the methodology used will appear shortly (A. Benedetto and D. Gems in preparation).

Protein oxidation measurements

Protein oxidation was measured using the OxyBlot Protein Oxidation Detection Kit (Merck Millipore). For analysis of mutant lines, 20 worms were harvested in 20μl cell lytic (Sigma) supplemented with 50mM DTT and protease inhibitor cocktail (Roche). For analysis of RNAi samples, protein extracts were made from synchronised plates of worms fed control or *skn-1* RNAi. Adult worms on day 1 of adulthood were used in both cases. Samples were kept on ice. Samples were sonicated for 5 minutes at 30 second intervals using a Bioruptor sonicator (Diagenode), debris spun down and 5μl protein extract transferred to a new tube. The carbonyl groups in each sample were derivatized to 2,4-dinitrophenylhydrazone (DNP-hydrazone), the modified samples separated by SDS PAGE, transferred to a membrane and western blotted using a primary antibody specific to the DNP moiety of the proteins. After analysis, blots were stripped and re-probed with a β-actin antibody (Santa Cruz) as a loading control. Imaging and quantification of bands was carried out using the ImageQuant LAS4000 imaging system and software (GE Healthcare).

RT qPCR and chromatin immunoprecipitation

RNA was isolated from adult worms after transfer of the worms to an unseeded NGM plate to remove *E. coli*. 50 - 100 worms were used for each assay. RNA was extracted using Trizol (Sigma) and cDNA synthesized using SuperScript II reverse transcriptase with oligo dT (Invitrogen). qRT-PCR was carried out using Fast SYBR Green Master Mix (Applied Biosystems) and the 7900 HT Fast PCR system (Applied Biosystems). Normalization of transcript quantity was carried out using the geometric mean of three stably expressed reference genes Y45F10D.4, *pmp*-3, and *cdc-42* in order to control for cDNA input, as previously described (Hoogewijs et al. 2008). Primer sequences to detect *skn-1* by qPCR were designed by Primerdesign (sequences available on request). Statistical analysis was preformed using a student t-test. The protocol for chromatin immunoprecipitation PCR was as described (Tullet et al. 2014) and the primer sequences as follows: *skn-1b/c* promoter F: gcgcgccgatagagtagatc R: ccctgcgtgtctacagtttcag; *skn-1b* promoter F: gcacgcctccttcattagtc R: gctggttgcactttctcctc; control region F: tgtatgggggtgaacaggat R: cccggagctcagactacatc.

Epifluorescence microscopy

Worms were raised at 15˚C, picked at L4 stage, and shifted to 25˚C for 24 hr to increase transgenic extrachromosomal array expression and to induce the *daf-2* phenotype in the *daf-2(e1370)* 1 day old adults. For each slide, 30-40 worms were mounted in M9 + 0.2% levamisole on a 2% agarose pad and imaged within 30 min. Quantification of GFP expression from transgenic strains was carried out using a Leica DMRXA2 microscope with a GFP filter cube (excitation: 470/40 nm; emission: 525/50 nm), an Orca C10600 digital camera (Hamamatsu) and Volocity image analysis software (Improvision).

Author contributions

Conceived and designed the experiments: DG, JMAT. Performed the experiments: JWG, CA, EC, AY, AB, MAT, AFG, KS, JMAT. Analyzed the data: DG, JMAT. Wrote the paper: DG, JMAT.

Table S1: Statistics for lifespan measurements

Trial number	Strain	Genotype and treatments	Mean life span	% Difference	N (total)	*p*
1	N2	Control RNAi	23.6	-	70 (97)	-
	N2	*daf-16* RNAi	18.9	-19.9[a]	89 (99)	<0.0001[a]
	TJ356	*daf-16(oe);* control RNAi	28.7	+21.6[a]	64 (67)	<0.0001[a]
	TJ356	*daf-16(oe); daf-16* RNAi	20.1	-14.8[a]	108 (108)	<0.0001[b]<br>NS[a *daf-16* RNAi]
1	N2	+	21.3	-	88 (95)	-
	EU31	*skn-1(zu135)*	18.2	-14.6[a]	19 (20)	0.0324[a]
	TJ356	*daf-16(oe)*	24.4	+14.6[a]	84 (90)	0.006[a]
	GA1026	*daf-16(oe); skn-1(zu135)*	25.0	+17.4[a]	44 (52)	0.0079[a]<br>0.5803[b]
2	N2	+	21.5	-	63 (74)	-
	EU31	*skn-1(zu135)*	16.3	-24.2[a]	32 (36)	<0.0001[a]
	TJ356	*daf-16(oe)*	24.2	+12.6[a]	98 (107)	0.0015[a]
	GA1026	*daf-16(oe); skn-1(zu135)*	22.8	+6.0[a]	59 (60)	0.1695[a]<br>0.2388[b]
3	N2	+	15.7	-	82 (98)	-
	EU31	*skn-1(zu135)*	17.1	+8.9[a]	96 (96)	0.0003 [a]
	TJ356	*daf-16(oe)*	21.7	+38.2[a]	96 (96)	<0.0001[a]
	GA1026	*daf-16(oe); skn-1(zu135)*	29.6	+88.5[a]	95 (95)	<0.0001[a]<br><0.0001[b]
1	N2	Control RNAi	26.3	-	63 (100)	-
	N2	*skn-1* RNAi	23.2	-11.79[a control]	70 (100)	<0.0001[a control]
	TJ356	*daf-16(oe)*; control RNAi	34.1	+29.66[a control]	76 (100)	<0.0001[a control]
	TJ356	*daf-16(oe)*; *skn-1* RNAi	31.2	-8.50[b control]	72 (100)	<0.0001[a control]<br>0.03[b control]
2	N2	Control RNAi	26.4		67 (100)	
	N2	*skn-1* RNAi	22.3	-15.53[a control]	73 (100)	<0.0001[a control]
	TJ356	*daf-16(oe)*; control RNAi	33.7	+27.65[a control]	78 (100)	<0.0001[a control]
	TJ356	*daf-16(oe)*; *skn-1* RNAi	31.2	-7.42[b control]	73 (100)	<0.0001[a control]<br>0.01[b control]
3	N2	Control RNAi	21.9	-	68 (85)	-
	N2	*skn-1* RNAi	20.5	-6.4 [a control]	83 (85)	0.0112[a control]
	TJ356	*daf-16(oe)*; control RNAi	23.3	+6.0[a control]	101 (110)	0.0017[a control]
	TJ356	*daf-16(oe)*; *skn-1* RNAi	23.2	-0.4 [b control]	76 (80)	0.0115[a control]<br>NS[b control]
1	N2	Control RNAi	26.3	-	63 (100)	-
	N2	*skn-1* RNAi	23.2	-11.40 [a control]	70 (100)	<0.0001[a control]
	GA1066	*daf-16(oe)*; control RNAi	31.5	+19.77 [a control]	59 (80)	<0.0001[a control]
	GA1066	*daf-16(oe)*; *skn-1* RNAi	29.9	-5.08 [b control]	68 (100)	<0.0001[a control]<br>NS[b control]
	GA1064	*Pges-1::daf-16(oe)*; control RNAi	29.2	+11.03 [a control]	48 (100)	<0.0001[a control]
	GA1064	*Pges-1::daf-16(oe)*; *skn-1* RNAi	28.2	- 3.42 [b control]	59 (100)	<0.0001[a control]<br>0.02[b control]

2	N2	Control RNAi	26.4	-	67 (100)	-
	N2	*skn-1* RNAi	22.3	-15.53 [a control]	73 (100)	<0.0001[a control]
	GA1066	*daf-16(oe)*; control RNAi	30.3	+14.77 [a control]	48 (100)	<0.0001[a control]
	GA1066	*daf-16(oe)*; *skn-1* RNAi	29.3	-3.30 [b control]	58 (100)	<0.0001[a control]<br>NS [b control]
	GA1064	*Pges-1::daf-16(oe)*; control RNAi	33.1	+25.38 [a control]	44 (100)	<0.0001[a control]
	GA1064	*Pges-1::daf-16(oe)*; *skn-1* RNAi	29.9	-3.30 [b control]	52 (100)	<0.0001[a control]<br>NS[b control]
1	N2	Control RNAi	21.7	-	72 (80)	-
	N2	*daf-2* RNAi	37.0	+70.5[a control]	65 (80)	<0.0001[a control]
	EU31	*skn-1(zu135);* control RNAi	21.4	-	59 (68)	NS[a control]
	EU31	*skn-1(zu135); daf-2* RNAi	33.0	+54.2[c control]	60 (80)	0.0091[a *daf-2* RNAi]
	TJ356	*daf-16(oe);* control RNAi	26.6	-	51 (80)	-
	TJ356	*daf-16(oe); daf-2* RNAi	33.2	+24.8[b control]	68 (79)	<0.0001[b control]<br>0.0002 [a *daf-2* RNAi]
	GA1026	*daf-16(oe); skn-1(zu135);* control RNAi	26.3	-	64 (80)	<0.0001 [a control]<br>NS[b control]
	GA1026	*daf-16(oe); skn-1(zu135); daf-2* RNAi	34.4	+31.0[d control]	32 (80)	NS [a *daf-2* RNAi]<br>NS[b *daf-2* RNAi]
2	N2	Control RNAi	17.6		98 (111)	
	N2	*daf-2* RNAi	29.1	+65.0[a control]	184 (196)	<0.0001[a control]
	EU31	*skn-1(zu135);* control RNAi	14.5		200 (205)	<0.0001[a control]
	EU31	*skn-1(zu135); daf-2* RNAi	22.0	+52.0[c control]	195 (210)	<0.0001[a *daf-2* RNAi]
	TJ356	*daf-16(oe);* control RNAi	20.7		80 (87)	
	TJ356	*daf-16(oe); daf-2* RNAi	25.5	+23.2[b control]	25(28)	<0.0001[a *daf-2* RNAi]
	GA1026	*daf-16(oe); skn-1(zu135);* control RNAi	20.5	-	125 (133)	0.001[a control]<br>NS[b control]
	GA1026	*daf-16(oe); skn-1(zu135); daf-2* RNAi	24.6	+20.0[d control]	108 (118)	<0.0001[a *daf-2* RNAi]<br>NS[b *daf-2* RNAi]
1	N2	No FUDR	21.6	-	31 (80)	-
	N2	FUDR	21.8	+0.9[a no FUDR]	65 (81)	NS[a no FUDR]
	EU40	*skn-1(zu129)* No FUDR	10.7	-	55 (60)	-
	EU40	*skn-1(zu129)* FUDR	12.2	+14.0 [c no FUDR]	52 (60)	0.0264 [c no FUDR]
	EU31	*skn-1(zu135)* No FUDR	12.0	-	76 (80)	-
	EU31	*skn-1(zu135)* FUDR	15.2	+26.7 [c no FUDR]	78 (81)	<0.0001 [c no FUDR]
2	N2	No FUDR	20.6	-	49 (60)	-
	N2	FUDR	22.0	+6.8 [a no FUDR]	87 (100)	0.0256 [a no FUDR]
	EU40	*skn-1(zu129)* No FUDR	12.7	-	44 (80)	-
	EU40	*skn-1(zu129)* FUDR	13.5	+6.3 [c no FUDR]	77 (79)	NS [c no FUDR]
	EU31	*skn-1(zu135)* No FUDR	15.6	-	57 (62)	-
	EU31	*skn-1(zu135)* FUDR	17.3	+10.9 [c no FUDR]	95 (100)	0.0279 [c no FUDR]
	EU1	*skn-1(zu67)* No FUDR	14.1	-	66 (77)	-
	EU1	*skn-1(zu67)* FUDR	14.0	-0.7 [c no FUDR]	88 (98)	NS [c no FUDR]
1	N2	+	19.4		79 (89)	

	GA1001	*aak-2(ok524)*	15.8	-18.6[a]	70 (80)	<0.0001[a]
	TJ356	*daf-16(oe)*	22.5	+15.9 [a]	70 (80)	0.0033[a]
	GA1054	*daf-16(oe); aak-2(ok524)*	21.6	-4.0[b]	67 (80)	0.0320[a]<br>0.4857[b]
2	N2	+	18.6	-	74 (80)	-
	GA1001	*aak-2(ok524)*	14.2	-23.7[a]	69 (79)	<0.0001[a]
	TJ356	*daf-16(oe)*	21.4	+15.8[a]	65 (80)	0.0046[a]
	GA1054	*daf-16(oe); aak-2(ok524)*	19.2	-10.0[b]	63 (80)	0.9282[a]<br>0.0074[b]
3	N2	+	21.7	-	94 (100)	-
	GA1001	*aak-2(ok524)*	15.0	-30.9[a]	94 (100)	<0.0001[a]
	TJ356	*daf-16(oe)*	22.6	+4.0[a]	90 (99)	0.5158[a]
	GA1054	*daf-16(oe); aak-2(ok524)*	21.1	-6.6[b]	69 (80)	0.2955[a]<br>0.0247[b]

% difference in mean lifespan compared to N2.
N, number of worm deaths (total worms scored including those censored).
p, probability (determined by log rank test) of being the same as specified control: [a]N2, [b]TJ356, [c]*skn-1* or [d]*skn-1(zu135) zIs356 [DAF-16::GFP]* (appropriate control allele used in each case). NS, not statistically significant (p>0.05).

Supplementary figure legends

Fig S1. Resistance to heat stress measured in liquid. SKN-1 is not required for the increased resistance of *daf-16(oe)* worms to heat.

Fig S2. *skn-1* mutation does not affect the germline hyperplasia and basal membrane disruption of the germline of *daf-16(oe)* animals. (A) Images from Nomarski microscopy showing the germline phenotype of *daf-16(oe)* and *daf-16(oe); skn-1* animals. Young adult animals were examined at 100X magnification and scored for the presence of germ cells outside the basal membrane of the germline (indicated by arrows). This is indicative of the basal membrane being disrupted. This was not observed in any N2 or *skn-1(zu135)* animals examined. (B) Quantification of penetrance of phenotype. Mean ± S.D. of at least 3 trials.

Fig S3. Tests for interactions between factors affecting lifespan. (A) The lifespan of different *skn-1* mutants is differentially affected by FUDR (trial 1 in Table S1). FUDR caused a slight increase in the mean lifespan of *skn-1(zu135)* mutants (p=0.02) but this strain was still markedly short lived compared to wild type. No lifespan extension by FUDR was observed in other *skn-1* mutants (Table S1). (B) The effect of *skn-1* on *daf-16(oe)* longevity is not affected by *daf-2* RNAi (trial 1 in Table S1). Trials conducted at 20˚C, with 40μM FUDR. (C) *daf-16(oe)* Age does not require AAK-2 (AMP kinase)(trial 1 in Table S1). Trials conducted at 20˚C, with 80μM FUDR. Similarly, RNAi of the AMPK γ subunit genes *aakg-4* and *aakg-5,* which partially suppress *daf-2* longevity (Tullet et al. 2014), did not suppress *daf-16(oe)* Age (data not shown).

Fig S4. No difference in protein damage detected in response to *skn-1* mutation or *skn-1* RNAi in N2 or *daf-16(oe)* animals. (A, B) Quantification of protein oxidation determined using Oxyblots. Mean ± S.D. of at least 3 trials. There was no significant differences found between any of the samples. *skn-1(zu135)* and *zIs356 [Pdaf-16::DAF-16a::GFP; rol-6(su1006)]* were used.

Fig S5. *skn-1* mRNA and *Pskn-1b/c::GFP* fluorescence levels. (A) Endogenous *skn-1c* and *skn-1b* mRNA levels are not dependent on IIS. Mean ± S.D. from 4 trials. Thus, using qRT-PCR to compare *skn-1* mRNA levels in *daf-2(e1370)* and *daf-16(mgDf50); daf-2* we were unable to verify previous microarray data (McElwee et al. 2007). We mined other available *daf-2* vs *daf-16; daf-2* microarray, RNA Seq and proteomic data sets (Tepper et al. 2013; Dong

et al. 2007; Riedel et al. 2013; Troemel et al. 2006; Budovskaya et al. 2008) but could find no further evidence of up-regulation of *skn-1* by rIIS. (B) *Pskn-1::GFP* was expressed in mesendodermal tissues.(C) No increase in GFP fluorescence in worms expressing the *Pskn-1b/c::gfp* reporter in *daf-2* relative to *daf-16; daf-2*. The *Pskn-1b/c::gfp* reporter was remade using specifications as previously described (An & Blackwell 2003). The same result was obtained using a second *Pskn-1b/c::gfp* transgene array (not shown). Means ± S.D. from 3 independent trials.

Supplementary References

An JH & Blackwell TK (2003) SKN-1 links C. elegans mesendodermal specification to a conserved oxidative stress response. *Genes Dev.* 17, 1882–1893.

Brenner S (1974) The genetics of Caenorhabditis elegans. *Genetics* 77, 71–94.

Budovskaya Y V, Wu K, Southworth LK, Jiang M, Tedesco P, Johnson TE & Kim SK (2008) An elt-3/elt-5/elt-6 GATA transcription circuit guides aging in C. elegans. *Cell* 134, 291–303.

Coburn C, Allman E, Mahanti P, Benedetto a, Cabreiro F, Pincus Z, Matthijssens F, Araiz C, Mandel a, Vlachos M, Edwards S a, Fischer G, Davidson a, Pryor RE, Stevens a, Slack FJ, Tavernarakis N, Braeckman BP, Schroeder FC, Nehrke K & Gems D (2013) Anthranilate fluorescence marks a calcium-propagated necrotic wave that promotes organismal death in C. elegans. *PLoS Biol.* 11, e1001613.

Dong MQ, Venable JD, Au N, Xu T, Park SK, Cociorva D, Johnson JR, Dillin A & Yates 3rd JR (2007) Quantitative mass spectrometry identifies insulin signaling targets in C. elegans. *Science (80-.).* 317, 660–663.

Hoogewijs D, Houthoofd K, Matthijssens F, Vandesompele J & Vanfleteren JR (2008) Selection and validation of a set of reliable reference genes for quantitative sod gene expression analysis in C. elegans. *BMC Mol Biol* 9, 9.

Hsin H & Kenyon C (1999) Signals from the reproductive system regulate the lifespan of C. elegans. *Nature* 399, 362–366.

McElwee JJ, Schuster E, Blanc E, Piper MD, Thomas JH, Patel DS, Selman C, Withers DJ, Thornton JM, Partridge L & Gems D (2007) Evolutionary conservation of regulated longevity assurance mechanisms. *Genome Biol* 8, R132.

Riedel CG, Dowen RH, Lourenco GF, Kirienko N V, Heimbucher T, West JA, Bowman SK, Kingston RE, Dillin A, Asara JM & Ruvkun G (2013) DAF-16 employs the chromatin remodeller SWI/SNF to promote stress resistance and longevity. *Nat. Cell Biol.* 15, 491–501.

Tepper RG, Ashraf J, Kaletsky R, Kleemann G, Murphy CT & Bussemaker HJ (2013) PQM-1 complements DAF-16 as a key transcriptional regulator of DAF-2-mediated development and longevity. *Cell* 154, 676–690.

Troemel ER, Chu SW, Reinke V, Lee SS, Ausubel FM & Kim DH (2006) p38 MAPK regulates expression of immune response genes and contributes to longevity in C. elegans. *PLoS Genet* 2, e183.

Tullet JM a (2014) DAF-16 target identification in C. elegans: past, present and future. *Biogerontology*.

Tullet JM, Hertweck M, An JH, Baker J, Hwang JY, Liu S, Oliveira RP, Baumeister R & Blackwell TK (2008) Direct inhibition of the longevity-promoting factor SKN-1 by insulin-like signaling in C. elegans. *Cell* 132, 1025–1038.

Tullet JMA, Araiz C, Sanders MJ, Au C, Benedetto A, Papatheodorou I, Clark E, Schmeisser K, Jones D, Schuster EF, Thornton JM & Gems D (2014) DAF-16/FoxO Directly Regulates an Atypical AMP-Activated Protein Kinase Gamma Isoform to Mediate the Effects of Insulin/IGF-1 Signaling on Aging in Caenorhabditis elegans. *PLoS Genet.* 10.
